# Annotating Spike Protein Polymorphic Amino Acids of Variants of SARS-CoV-2, Including Omicron

**DOI:** 10.1155/2022/2164749

**Published:** 2022-04-11

**Authors:** Gusti Ngurah Mahardika, Nyoman B. Mahendra, Bayu K. Mahardika, Ida B. K. Suardana, Made Pharmawati

**Affiliations:** ^1^The Animal Biomedical and Molecular Biology Laboratory, Udayana University, Jl. Sesetan-Markisa 6A, Denpasar 80223, Bali, Indonesia; ^2^The Department of Obstetrics and Genecology, The Faculty of Medicine, Udayana University, Denpasar, Bali, Indonesia; ^3^The Animal Biomedical and Molecular Biology Laboratory, Udayana University, Jl. Sesetan-Markisa 6A, Denpasar 80223, Bali, Indonesia; ^4^Virology Laboratory, The Faculty of Veterinary Medicine, Udayana University, Denpasar, Bali, Indonesia; ^5^The Biology Study Program, The Faculty of Mathematic and Natural Science, Udayana University, Kampus Bukit Jimbaran, Badung, Bali, Indonesia

## Abstract

The prolonged global spread and community transmission of severe acute respiratory syndrome virus 2 (SARS-CoV-2) has led to the emergence of variants and brought questions regarding disease severity and vaccine effectiveness. We conducted simple bioinformatics on the spike gene of a representative of each variant. The data show that a number of polymorphic amino acids are located mostly on the amino-terminal side of the S1/S2 cleavage site. The Omicron variant diverges from the others, with the highest number of amino acid substitutions, including the receptor-binding site (RBS), epitopes, S1/S2 cleavage site, fusion peptide, and heptad repeat 1. The current sharp global increase in the frequency of the Omicron genome constitutes evidence of its high community transmissibility. In conclusion, the proposed guideline could give an immediate insight of the probable biological nature of any variant of SARS-Cov-2. As the Omicron diverged the farthest from the original pandemic strain, Wuhan-Hu-1, we expect different epidemiological and clinical patterns of Omicron cases. On vaccine efficacy, slight changes in some epitopes while others are conserved should not lead to a significant reduction in the effectiveness of an approved vaccine.

## 1. Introduction

The emergence of various variants of the severe acute respiratory syndrome virus 2 (SARS-CoV-2) has led to questions about disease severity and vaccine effectiveness. As of 19 December 2021, over 273 million cases and over 5.3 million deaths have been reported globally (https://www.who.int). The World Health Organization defines SARS-CoV-2 variants as variants of concern (VOCs), variants of interest (VOIs), and variants under monitoring (VUMs). The VOCs listed in the GISAID database, which was accessed on December 26, 2021, are Omicron, Delta, Alpha, Beta, and Gamma; Lambda and Mu are VOIs; and GH/490R is a VUM.

The impact of amino acid substitutions in the variants must depend on their genetic make-up. The SARS-CoV-2 belongs to the lineage B (sarbecovirus) of *β*-CoVs of the *Coronaviridae* family, which is enveloped with single-stranded positive-sense RNA [1]. The genome of SARS-CoV-2 is more than 29 kb, which encodes for structural proteins of phosphorylated nucleocapsid (N) protein, spike glycoprotein (S), hemagglutinin-esterase (HE), membrane (*M*) protein, and envelope (*E*) protein, as well as nonstructural proteins of ORF1a, ORF1b, ORF3a, ORF3b, ORF6, ORF7a, ORF7b, ORF8, ORF9a, ORF9b, and ORF10 [1, 2]. ORF1a and 1b are also translated following -1 ribosomal frameshifting to produce ORF1ab protein [3]. Proteins translated from ORF1a, 1b, and 1ab form viral polymerase complex [4], while proteins of other ORF's are also known as accessory proteins [5]. However, it is generally believed that the spike protein is a major pathogenic coronavirus determinant. This surface protein possesses major immunogenic domains, and most gene-based vaccines target only the spike gene of SARS-CoV-2. The protein is highly glycosylated and cysteine rich, with two cleavage sites: S1/S2 and S2′ [6]. The glycosylation pattern of the SARS-CoV-2 spike involves N-linked and O-linked glycosylation [7]. Spike also has two protease cleavage sites, which are critical for virus activation and replication. Protease cleavage of the spike has been established as a critical determinant of coronavirus tropism and pathogenesis [8].

The epitopes of the spike protein seem to be linear and conformational. One group [9] mapped nine linear epitopes along the spike protein designated IdA-IdI; another group [10] identified 16 epitopes. Some epitopes overlap. Due to folding and trimerization, conformational epitopes in the spike protein of SARS-CoV-2 have been predicted [11].

The biological consequences of each variant are poorly understood. In this study, we performed functional annotation of amino acid changes in the spike protein of variants based on existing knowledge of coronaviruses as well as rapidly accumulating knowledge about SARS-CoV-2.

## 2. Materials and Methods

The sequence of the original SARS-CoV-2 strain of Wuhan-Hu-1 (Accession Number NC_045512) was downloaded from GenBank. The open reading frame (ORF) of the spike protein was selected as determined in the database. Ten complete sequences of each annotated variant were selected randomly from GISAID and downloaded. Using the spike gene of Wuhan-Hu-1, the first 15 nucleotides of the 5′-terminus were searched, and the sequence prior to the marked sequence was deleted. The last 15 nucleotides of Wuhan-Hu-1 were used to mark the 3′-end of the sequence, and all nucleotides after that marked sequence were deleted. We then manually selected the sequence data without any undefined or any “N” residue. When there was no “clean” sequence data for each variant, we evaluated another set of variant data. The selected sequences were translated into amino acid sequences and aligned using MEGA-X software [12]. Using the same software, the data were exported in Mega format and analyzed further for polymorphic or variable amino acids. The evolutionary history of variants was inferred using the neighbor-joining method [13]. Evolutionary distances were computed using the Kimura 2-parameter method [14]. The probable biological function of each residue was annotated using the guidelines shown in Supplementary Material 1.

## 3. Results

The dataset containing the representative of each variant used is available in Supplementary Material 2. Polymorphic amino acid residues of the spike protein of SARS-CoV-2 Wuhan Hu-1 and all variants are presented in Table 1. The data show that a number of polymorphic amino acids are located in the S1 domain, on the N-terminal side of the S1/S2 cleavage site. The number of polymorphic amino acids in this region is 73; the S2 domain has only 15. Glycosylation motif loss (GML) occurs once in Delta due to the T19R substitution and in Lambda due to the T76I substitution. Additional glycosylation motif (AGM) gain occurred twice in the Gamma variant, i.e., T20N and R191S, and once in Lambda, i.e., R249N. Cysteine residue loss (CRL) occurred once in the GH/490 variant due to the deletion of C136. Ten residues of the receptor binding site (RBS) are polymorphic, with only a single residue difference from Wuhan-Hu-1 in the Alpha and GH variants; the other variants, except for Omicron, carry two residues, and Omicron shows nine amino acid differences from Wuhan-Hu-1. The number of amino acid substitutions in various linear epitopes is 19; that of probable conformational epitopes is 16. The number of amino acid changes from Wuhan-Hu-1 at the mapped linear epitopes of various variants is 2, 6, 4, 2, 3, 3, 10, and 3 for Alpha, Beta, Gamma, Delta, Lambda, Mu, Omicron, and GH/490R, respectively. A single amino acid difference from Wuhan-Hu-1 at probable conformational epitopes occurred in Beta, Gamma, Delta, Mu, and GH/490R; three changes in Alpha, and seven each occurred in Lambda and Omicron. At the S1/S2 cleavage site of the Alpha, Beta, Delta, Lambda, and GH/490R variants differ from Wuhan-Hu-1 in one residue, with Omicron differing in two. All residues at this site have changed from the nonbasic amino acids Q/N/P to the basic amino acids H/R/K. At the fusion peptide site, only a single amino acid substitution occurred in Omicron. In heptad repeat 1 (HR1), a single amino acid alteration occurred in the Alpha, Gamma, and Mu variants, with Omicron displaying three alterations. In heptad repeat 2 (HR2) and the transmembrane domain, a single amino acid change occurred in the Gamma variant only.

**Figure 1:**
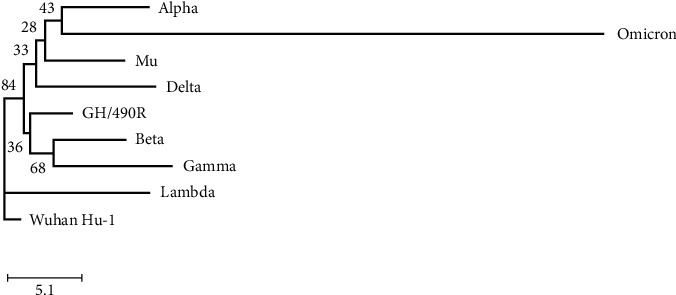
Evolutionary relationships of variants of SARS-CoV-2. The evolutionary history was inferred using the neighbor-joining method [13]. The evolutionary distances were computed using the Kimura 2-parameter method [14]. Evolutionary analyses were conducted in MEGA X [12]. The tree was rooted to Wuhan-Hu-1.

The phylogenetic analysis presented in Figure 1 shows two clusters of variants, with good bootstrap support of 88%. The Alpha, Delta, Mu, and Omicron variants form one cluster, and GH/490R, Beta, and Gamma form another. Lambda appears to have emerged directly from Wuhan-Hu-1. In the first cluster, Omicron forms a long branch from the other members of the group.

## 4. Discussion

The number of whole-genome sequence data for SARS-CoV-2 submitted to international databases poses a major computational challenge to obtain an indication of the possible impact of each strain before clinical and experimental data are available, especially in resource-limited countries. At the time of writing of this paper, the number of submitted whole-genome SARS-CoV-2 sequences was approximately 6.5 million. Here, we offer a simple bioinformatic protocol for gene mining and predicting the possible biological meaning of genetic changes in strains. The GISAID initiative has enabled data mining by providing a variant tag for each submitted sequence. We proposed a guideline of the probable biological function of each residue in the spike protein of SARS-Cov-2 based on current knowledge which could be adjusted following the fast flow of upcoming research reports.

It is generally believed that the phenotypic nature of viruses is mostly polygenic, whereby the entire genetic composition of strains determines the biology of the virus. This should also be true for coronaviruses, including SARS-CoV-2. The mechanism of pathogenesis of the Middle East severe acute respiratory syndrome coronavirus (MERS-CoV) mainly occurs through interaction between spike and cellular receptors, papain-like protease PLpro, and accessory proteins such as p4a and membrane *M* protein [15]. For SARS-CoV-2, various nonstructural proteins, such as PLpro [16] and various accessory proteins [5], have been described as contributing to virus biology and pathogenesis. However, focusing on the spike gene is also important, as a body of literature provides evidence on the key functions of this protein. The S1 domain, which carries major antigenic determinants, mediates receptor recognition and viral attachment to initiate host cell entry [17]. The NTD domain contributes to the host range [18]. Binding of the receptor-binding domain to receptors initiates infection [19], and the S2 domain mediates membrane fusion [17, 20, 21].

Various polymorphic amino acids are located mostly in the S1 domain, downstream from the S1/S2 cleavage site. The number of polymorphic amino acids at this site is 73, with only 15 in the S2 domain. We believe that as the S1 domain of the spike is located on the surface of the virion, thus allowing many substitutions, but that the S2 domain must be conserved to preserve virus integrity. Glycosylation and cysteine residues are also crucial for maintaining virus integrity. Our data show that glycosylation motif loss occurred once in the Delta and Lambda variants and that additional glycosylation motif gain occurred twice in Gamma and once in Lambda. Cysteine residue loss (CRL) occurred only once in the GH/490R variant.

The newest variant, Omicron, which was recently identified, exhibits nine amino acid changes in the RBD, whereas other variants are more conserved, with only one or two residue differences from Wuhan-Hu-1. Therefore, it is plausible to expect biological changes in Omicron that differ from those of other variants as well as the original strain. Tracking variant occurrence on the GISAID website revealed a sharp global increase from under 1% on November 29, 2021, to 50% of circulating strains on December 29, 2021. Higher transmissibility is evident.

The Omicron linear and conformational epitopes diverge most from the Covid-19 origin strain Wuhan-Hu-1, with ten and seven amino acid substitutions, respectively. Seven amino acid substitutions at conformational epitopes also occur in the Lambda variant. The other variants have only 2–6 amino acid changes in linear and 1–3 substitutions in conformational epitopes of spike. The identified SARS-CoV-2 spike epitopes consist of at least 10–20 residues or longer. As MHC-1-presenting B- and T cell epitopes are limited to 9–11 residues and MHC-II-presenting epitopes are limited to 9–22 [22], the identified epitopes of the spike protein of SARS-CoV-2 need to be refined. Moreover, due to the multiple epitopes of more than 20, a slight change at some epitopes while others are conserved should not lead to a significant reduction in the effectiveness of existing vaccines. Reports on the reduced efficacy of vaccines against variants based on in vitro experiments [23] should not cause concern, as they might not significantly reduce vaccine efficacy in vivo. Indeed, the immune system consists of an array of components, such as cytokines, complement activation, and macrophage opsonization [24–26], as already reported in SARS-CoV-2 [27–30]. Moreover, cellular mediated immunity must pose critical role in a complete immune protection in SARS-CoV-2 [31], which is not involved in an in vitro neutralization testing.

The S1/S2 cleavage site changes from nonbasic Q/N/P to basic H/R/K amino acids might be critical to the biology of the virus, especially Omicron. Although other variants show a single amino acid change, Omicron exhibits two. Moreover, all variants carry more basic S1/S2 cleavage sites, and Omicron has the most basic S1/S2 cleavage site. It is well-documented for influenza viruses that the polybasic cleavage site allows for ubiquitous cellular protease activation for the virus to initiate infection [32–34]. The cleavage site of the origin of SARS-CoV-2 is indeed polybasic with an NH-PRRAR-COOH motif. A P681H change occurred in Alpha, Mu, and Omicron, with P681R in delta and GH/490R. A more basic cleavage site was acquired in Omicron due to the N679K substitution. For the Delta variant, the more basic cleavage site might have contributed to its transmissibility and clinical outcomes [35, 36]. Therefore, it is plausible to expect higher transmissibility of Omicron as its cleavage site is more basic than that of the delta variant. However, its clinical consequences among nonimmune people, who are unvaccinated or have not experienced natural infection, are expected to be reported soon.

Despite the protein changes of Omicron, it has a relatively conserved S2 domain. Omicron shows amino acid alterations in the fusion peptide and HR1, with one and three substitutions, respectively, which might have an impact on fusion capability. As described previously, these domains mediate membrane fusion in infected cells [17, 20, 21].

Phylogenetic analysis showed that the Omicron forms a long branch from the other members of the cluster. This phenomenon is most frequently observed due to isolation and having no known close relatives [37]. The ancestor of this variant might have been circulating without notice, or the number of genome sequences from the area of circulation was limited. Another explanation is that dramatic changes might have occurred shortly before its identification.

We expect some biological changes due to amino acid substitution of the spike protein of SARS-CoV-2, especially for omicron. The most significant number of amino acid substitutions occurred in Omicron, with divergence in the RBS, epitopes, S1/S2 cleavage site, fusion peptide, and HR1. The sharp global increase in dominance of 50% of circulating viruses shows evidence of their high community transmissibility. As multiple epitopes of more than 20 residues exist on spike, a slight change in some epitopes while others are conserved should not lead to a significant reduction in existing vaccine effectiveness. The emergence of Omicron with dramatic changes and an unknown recent ancestor should draw attention from the global community for resource mobilization.

In conclusion, the proposed guideline could give an immediate insight into the probable biological nature of any variant of SARS-Cov-2. The Omicron diverged the farthest from the original pandemic strain Wuhan-Hu-1 with divergence in the RBS, epitopes, S1/S2 cleavage site, fusion peptide, and HR1. Therefore, we expect different epidemiological and clinical pattern of Omicron cases. On the vaccine efficacy, slight changes in some epitopes while others are conserved should not lead to a significant reduction in existing vaccine effectiveness.

## Figures and Tables

**Table 1:** Polymorphic amino acids residues of spike protein of SARS-CoV-2 Wuhan Hu-1 and all variants with possible biological function.

Amino acid position^*∗*^	SARS-CoV-2 variant									Known Function/probable biological impact^*∗∗*^
	Wuhan Hu-1	Alpha	Beta	Gamma	Delta	Lambda	Mu	Omicron	GH/490R	
9	P								L	SP
18	L		F	F						NTD
19	T				R					NTD; GML
20	T			N						NTD; AGM
26	P			S						NTD; IdA; PCE
67	A							V		NTD
69	H	Del						Del		NTD
70	V	Del						Del		NTD; PCE
75	G					V				NTD
76	T					I				NTD; GML
80	D		A							NTD
95	T				I		I	I		NTD
136	C								Del	NTD; CRL
137	N								Del	NTD
138	D			Y					Del	NTD
139	P								Del	NTD
140	F								Del	NTD
141	L								Del	NTD
142	G				D			D	Del	NTD
143	V	Del						Del	Del	NTD
144	Del	Del	Del	Del	Del	Del	T	Del	Del	NTD
145	Y	V					S	Del	Del	NTD
146	Y						N	Del		NTD
154	M				T					NTD
157	E				G					NTD
158	F				Del					NTD
159	R				Del					NTD
191	R			S						NTD; AGM
212	N							I		NTD; PCE
213	L							V		NTD; PCE
214	V							R		NTD; PCE
215	R							E		NTD
216	Del	Del	Del	Del	Del	Del	Del	P	Del	NTD
217	Del	Del	Del	Del	Del	Del	Del	E	Del	NTD
218	D		G						G	NTD
244	L		Del							NTD; IdB
245	L		Del							NTD; IdB
246	A		Del							NTD; IdB
248	H								P	NTD; IdB
249	R					N				NTD; AGM
250	S					Del				NTD; PCE
251	Y					Del				NTD; PCE
252	L					Del				NTD; PCE
253	T					Del				NTD; PCE
254	P					Del				NTD; PCE
255	G					Del				NTD; PCE
256	D					Del				NTD; PCE
342	G							D		RBD; IdD
349	R						K			RBD; IdD
374	S							L		RBD
376	S							P		RBD
378	S							F		RBD; IdE/He4
420	K		N	T				N		RBD; He5
443	N							K		RBD; RBS
449	G							S		RBD; RBS
455	L				R	Q				RBD; RBS; IdF
480	S							N		RBD; RBS
481	T				K			K		RBD; RBS
487	E		K	K			K	A	K	RBD; RBS; IdG
493	F					S				RBD; RBS; IdG
496	Q							R		RBD; RBS; IdG
499	G							S		RBD; RBS; IdG
501	Q							R		RBD; RBS; IdG
504	N	Y	Y	Y			Y	Y		RBD; RBS
508	Y							H		RBD
550	T							K		IdH/He6-7
573	A	D								IdH/He6-7
617	D	G	G	G	G	G	G	G	G	IdH/He6-7
658	H			Y				Y		
678	Q					H				S1/S2-CS
682	N							K		S1/S2-CS; PCE
684	P	H			R		H	H	R	S1/S2-CS; PCE
693	Q		H							S1/S2-CS; PCE
704	A		V							
719	T	I								GML
767	N							K		He9-11
799	D							Y		FP
858	N							K		
862	T					N				
953	D				N		N			HR1
957	Q							H		HR1
972	N							K		HR1
984	L							F		HR1; PCE
985	S	A								HR1; PCE
1023	A						S			
1030	T			I						
1121	D	H								
1179	V			F						HR2; TM

^*∗*^The positions were determined after alignment of all variants as available at supplementary material. Numbering 1–143 is equal to residues no. 1–143 of Wuhan-Hu-1. Number 144–215 is Wuhan-Hu-1 plus 1. Number >215 become Wuhan-Hu-1 plus 3; ^*∗∗*^SP: signal peptide; NTD: N-terminal domain of S1; S1/S2 CS: S1/S2 cleavage site; RBD: receptor binding domain; RBS: receptor binding site; FP: fusion peptide; HR1 or HR2: heptad repeat 1 or 2; TM: transmembrane; IdA, IdB, IdC, IdD, IdE/He4, IdF, IdG, IdH/He6-7, IdI/He12-13, He1, He2-3, He5, He8, He9-11, He14, He15, and He16: corresponding linear epitopes as described in Supplementary Material 1; PCE: probable conformational epitopes; GML: glycosylation motive loss; AGM: additional glycosylation motive; CRL: cysteine residue loss.

## Data Availability

All the data are provided in the text as well as in the supplementary materials.

## Abbreviations

AGM: Additional glycosylation motive
CRL: Cysteine residue loss
FP: Fusion peptide
GML: Glycosylation motive loss
HR1 or HR2: Heptad repeat 1 or 2
NTD: N-terminal domain of S1
PCE: Probable conformational epitopes
RBD: Receptor binding domain
RBS: Receptor binding site
S1/S2 CS: S1/S2 cleavage site
SARS-CoV-2: Severe acute respiratory syndrome coronavirus 2
SP: Signal peptide
TM: Transmembrane
VOC: Variant of concern
VOI: Variant of interest
VUM: Variant under monitoring.

## Additional Points

*Statement of significance of the study*. The number of whole-genome sequence data for SARS-CoV-2 submitted to international databases poses a major computational challenge to obtain an indication of the possible impact of each strain before clinical and experimental data are available, especially in resource-limited countries. Here, we offer a simple bioinformatic protocol for gene mining and predicting the possible biological meaning of genetic changes in strains. We proposed a guideline of the probable biological function of each residue in the spike protein of SARS-Cov-2 based on current knowledge which could be adjusted following the fast flow of upcoming research reports. Applying the guideline to representatives of various SARS-CoV-2 variants, it is remarkable that polymorphic amino acids are located mostly on the amino-terminal side of the S1/S2 cleavage site. The Omicron variant diverges from the others at including the receptor-binding site (RBS), epitopes, S1/S2 cleavage site, fusion peptide, and heptad repeat 1. The unique RBS and S1/S2 cleavage sites could contribute to the sharp global increase of Omicron cases. However, a slight change in some epitopes while others are conserved should not lead to a significant reduction in the effectiveness of existing vaccines.

## Supplementary Materials

Supplementary Materials Supplementary Materials. The probable biological function of each residue was annotated using the guidelines shown in Supplementary Material 1. The dataset containing the representative of each variant used is available in Supplementary Material 2.
