# Supplementary material for: Annotating Spike Protein Polymorphic Amino Acids of Variants of SARS-CoV-2, Including Omicron
Source: Biochem Res Int. 2022 Apr 11;2022:2164749. doi: 10.1155/2022/2164749 (PMC9017565; doi:10.1155/2022/2164749)
Supplement: Supplementary Materials — The probable biological function of each residue was annotated using the guidelines shown in Supplementary Material 1. The dataset containing the representative of each variant used is available in Supplementary Material 2. [file 2164749.f1.zip › 2164749.f1/Supplementary Material 1.docx]

Supplementary Material 1

Known biological function of various domains of the spike protein of SARS-CoV-2 or other Coronaviruses

| Position* | Function | References |
| --- | --- | --- |
| 1-13 | Signal Peptide (SP) | [20]. |
| 13-305 | N-terminal domain (NTD) of S1 | [20]. |
| 671-694 | S1/S2 Cleavage Site | [8]. |
| 685-686 | S1/S2 Cleavage | [20]. |
| 815-816 | S2’ Cleavage | [20]. |
| 806-821 | S2’ Cleavage Site | [8, 20]. |
| 319-541 | Receptor Binding Domain (RBD) | [20]. |
| 437-508 | Receptor Binding Site (RBS)/Receptor Binding Motive (RBM) | [20]. |
| 788-806 | Fusion Peptide (FP) | [20] |
| 912-984 | Heptad Repeat 1 (HR1) | [20] |
| 1163-1213 | Heptad Repeat 2 (HR2), | [20] |
| 1213-1237 | Transmembrane (TM) | [20] |
| 1237 - end | Cytoplasmic Domain (CD) | [20] |
|  | Linear Epitope/Antibody Binding Sites (ABS)** |  |
| 21-45 | IdA | [9] |
| 221-245 | IdB | [9] |
| 261-285 | IdC | [9] |
| 330-349 | IdD | [9] |
| 375-394 | IdE/He4 | [9, 10] |
| 450-469 | IdF | [9] |
| 480-499 | IdG | [9] |
| 522-646 | IdH/He6-7 | [9, 10] |
| 902-926 | IdI/He12-13 | [9, 10] |
| 194-210 | He1 | [10] |
| 291-325 | He2-3 | [10] |
| 410-426 | He5 | [10] |
| 722-739 | He8 | [10] |
| 747-771 | He9-11 | [10] |
| 1101-1115 | He14 | [10] |
| 1129-1145 | He15 | [10] |
| 1213-1229 | He16 | [10] |
|  | Probable Conformational Epitopes (PCE) | [11] |
| 22-26 | PCE |  |
| 70 | PCE |  |
| 147-150 | PCE |  |
| 173-187 | PCE |  |
| 207-213 | PCE |  |
| 247-253 | PCE |  |
| 676-689 | PCE |  |
| 793-794 | PCE |  |
| 808-812 | PCE |  |
| 850-854 | PCE |  |
| 979-984 | PCE |  |
| 1099-1100 | PCE |  |
| 1139-1146 | PCE |  |
|  | N-Link (NLG) and O-Link Glycosylation (OLG) | [7] |
| N17XTT | NLG |  |
| SN61XT | OLG |  |
| SXTN74XT | NLG |  |
| N122XT | OLG |  |
| N149S | OLG |  |
| SXXN165XT | NLG |  |
| N234XT | OLG |  |
| N282XT | NLG |  |
| N331XT | NLG |  |
| N343XT | NLG |  |
| TN603TS | OLG |  |
| N616XT | OLG |  |
| N657XS | OLG |  |
| SN709XS | NLG |  |
| TN717XT | NLG |  |
| N801XS | NLG |  |
| N1074XTT | OLG |  |
| SN1098XT | OLG |  |
| N1134XT | NLG |  |
| N1158XTS | NLG |  |
| SXXN1173XS | NLG |  |
| N1194XS | NLG |  |

Note:

*The numbering is based on the amino acid sequence of spike protein of Wuhan-Hu-1; **We propose a denotation of He-1 to H16 epitopes described in reference He at al. [10] for simple explanation. Since some epitopes described by this group are overlapped, we propose the name to be single denotation, for example epitope 6 and 7 of He-et al [10] are overlapped, we name it as He6-7. Moreover, since some epitopes overlapped with those found by Zhang et al. [9], we propose the name as IdN/HeN. For example, the epitope IdE found in reference [9] is overlapped with He4 in reference [9, 10], so we name it as IdE/He4.
